# Collectanea: Pathology and Practice

**Published:** 1834-10-01

**Authors:** 


### COLLECTANEA.

### PATHOLOGY AND PRACTICE.

### OPERATION FOR IMPERFORATE ANUS.

M. Roux was called to see a new-born infant, who had no vestige of an anus in the perinæum, the rectum terminating in the urethra. The urethra did not open at its extremity, but beneath. There was a sort of erection, with hiccup and vomiting. The physician who was first called in had cut the membrane which obstructed the urethra, but had not ventured to attempt to find the anus. M. Roux, having placed the infant on the knees of an assistant, made an incision, eight inches long, in the skin from before backwards, in the situation which the anus ought to occupy, and discovered the fibres of the sphincter, which he separated by dissection. Having arrived at the *levator ani*, he separated its fibres likewise, continuing his course towards the coccyx, for fear of touching the bladder, and, above this last layer of muscles, he came upon a mass of cellular tissue, in which his finger recognised a soft and fluctuating projection. This was punctured with the bistoury, and gave exit to a large quantity of meconium. The puncture was then enlarged so as to permit the introduction of the first phalanx of the forefinger, and a large bougie, covered with cerate, was afterwards substituted, and allowed to remain. On the following days, the fæces were voided by the wound, and some also made their escape by the urethra. The incision was then enlarged in the direction of the coccyx, in order to permit the passage of the fæces, and, in consequence of this precaution, the artificial anus soon fulfilled all the functions of the natural one. The infant still lives, and enjoys good health.—*Archives Génèrales.*

### OF THE REMOTE OR PREDISPOSING CAUSES OF CARIES OF THE TEETH.

Although caries of the teeth does not appear to arise from causes which affect the general health, many persons of robust constitution being observed to be exceedingly prone to this disease, and others, although of a more delicate frame, are almost wholly free from it, yet it is undoubtedly materially influenced by predisposing causes.

The first of these, perhaps, is hereditary predisposition; for it will generally be remarked, that, if the parents have bad teeth, the malformation will, in all probability, be reproduced in their offspring; and it is by no means uncommon to find the teeth of different individuals of a family decaying in exactly the same way at corresponding periods of life.

Imperfect congenital formation is another of the principal predisposing causes of caries. This may proceed either from the rudimentary pulps having been in an unhealthy condition, or it may arise from the inactivity of the vessels by which the enamel and osseous substance were secreted. Either of these occurrences will impair the organization of the teeth; and, of course, render them more prone to disease.

Another predisposing cause of caries is the irritation produced on the constitution and general health of the child, by all those diseases incidental to infancy, which usually occur at the same time that the permanent teeth are in the act of being formed.

Febrile diseases, and all those affections that are attended with long confinement, dyspepsia and sedentary habits, often give rise to caries of the teeth. It has been remarked, that the disease is particularly apt to arise during the confinement attending on parturition.

But of all the injurious agents on the teeth, and predisposing causes to early decay, there is none, I believe, so frequently destructive as mercury. The effect of this medicine on the teeth is usually observed only at a more advanced period of life, when the operation of "the specific" on the mouth is considered as a criterion of its introduction into the system. But I am convinced that it is in earlier years that most mischief is done by mercury, and observe with regret, that the importance of this circumstance has not hitherto been sufficiently noticed by preceding authors. The large and continued doses of calomel which are so indiscriminately given to children, for almost every disease, either, by weakening the constitution, prevent the proper formation of the teeth, or, by the powerful stimulus which mercury always exerts upon the absorbent vessels, cause the absorption of their substance as soon as it is deposited. I am convinced that it is solely from this use, or rather abuse of calomel, that we must trace, not only the origin of that tendency to early and extensive caries, which so frequently terminates in the total and premature destruction of the teeth of youth, but also, that it is from the same source that those deficiencies of the enamel proceed, which are considered as unaccountable, and from which the teeth sustain irreparable injury.—*Jobson on the Teeth.*

[A very sensible, well-written treatise, though there are one or two minor points on which we would willingly break a lance with the author. He says, for instance, at page 115, that "all acids and acidulous fluids should be sedulously avoided." Surely he must mean to limit this prohibition to the stronger acids. Who would or could avoid vinegar and the acidulous fruits? Mr. Jobson is opposed, and rightly, to the authors who imagine the tartar collected on the teeth to be one of the chief causes of caries. The fact is, that this encrustation is found most frequently on those teeth which most rarely become carious, and *vice versâ*. We strongly recommend this work to our readers.—[Ed. *Med. Quart. Rev.*]

INSENSIBILITY OF THE EYE.

The eye being thus opened, and the eyelids retained asunder, the eye loses all the extreme sensibility with which it is endowed for its security, and preservation in its ordinary state. Public opinion, which on medical subjects is generally erroneous, although for the most part founded on professional authority, is in no instance more injurious than in relation to the eye. It pronounces it to be an organ of a very delicate nature, exquisitely sensible, requiring the greatest delicacy of touch, and the utmost nicety of management; which opinion some oculists formerly found it convenient to support, and which the public may still continue to believe, without any great disadvantage; but students in surgery must be taught otherwise. They must learn, that the eye is not a delicate organ; that it will suffer more comparative violence, with less injury, than any other of importance in the whole body; that, so far from being exquisitely sensible, it is, when exposed in a healthy state, nearly the reverse, only becoming permanently so on the occurrence of inflammation; and that the ablest and most successful operators are not apparently, although they are in reality, the most tender in their proceedings. The opinion of the exquisite sensibility of the eye has arisen from the pain which is felt on the admission of a small piece of dirt, or a fly, between the eyelids; but this occurs from a wise and preservative provision of nature, on account of the insensibility of the eyeball itself. Let the eyelid be raised, and the same piece of dust applied to the surface of the eye, no pain, and scarcely a sensation, will be produced: remove the piece of dirt, turn out the lid, and whilst it is retained everted, place the piece of dirt upon it, no greater sensation will be induced than is felt when it is applied to the eyeball. The inference is, that both surfaces, when touched separately, are nearly insensible to this species of irritation. But let the same piece of dirt be put between the eyelid and the eyeball, and the sensation produced is exquisitely painful. To give rise to this sensation it is necessary that the two surfaces should come in contact, and that the foreign body be grasped between them. If this were not the case, an irreparable injury would often occur to the transparent part of the eye before it would be observed; and if the raising of the lid and the separation of the surfaces did not nearly annul sensation, an operation could not be performed for cataract; for who could bear quietly the sensation which must arise from pushing a needle into the eye, if it were analogous to that arising from a fly or a dry solid substance between the eye and the lids? The experiment may be tried in a very simple and conclusive manner by any one on himself, by merely keeping the lids apart by an effort of the will, when the end of the finger may be placed boldly on the eyeball without any inconvenience. Inflammation, by enlarging the vessels, gives rise to pain in the same way, and the sensation is at first as if some extraneous matter were interposed between the lids. I was the first to mention this fact, and to explain why it was so, although, I dare say, every body knew it just as well as I did; the only difference between us all being, that I was not afraid to tell the truth.—*Guthrie on Extraction of the Cataract.*

### SUTURE OF THE PERINEUM.

M. Roux has communicated to the Royal Academy of Medicine the result of the seventh operation which he has performed to effect the reunion of the perineum. The laceration had existed for six years, and yet his success was more rapid and more complete than in any of the other cases. In fact, in the former ones, when the ligatures were removed, there always remained a small opening from the rectum into the vagina, which did not close till after the lapse of some time. In the present case the reunion was immediate, and the cure was complete as soon as the ligatures had been removed. The reunion in front, indeed, has been so great, that the vulva has been somewhat contracted.—*Archives Génèrales, Août.*

### METHOD OF MAKING THE INCISION IN EXTRACTION OF THE CATARACT.

The pupil being fully dilated (and it is of no trifling importance to know that it ought to be dilated), the eyelids being separated, the eye fixed, and the knife ready, the surgeon commences the incision of the cornea, by introducing it near its edge, or junction with the sclerotica. Some say one quarter of a line, some say half a line, others a line: the intention of all is the same, viz. that it may be near to the edge of the cornea, in order to admit of the opening in it being as large as possible; it ought not nevertheless to touch the sclerotica, as parts of the same kind unite more readily than those which are of a dissimilar nature; and it should also be so much above the plane of the iris, that it will pass readily across without touching it. The upper half of the cornea must be completely divided, and, if anything, rather more than less, so that the point of the knife must be entered at nearly the least possible distance below the horizontal, or, as it is sometimes pedantically called, the equatorial line of the eye.

The manner of entering the point of the knife is disputed. The cornea being composed of several layers, constituting a substance of a certain thickness, there is some danger of passing the knife between the layers, and not across the anterior chamber of the eye, in front of the iris, if care be not taken that the cornea is fairly penetrated in the very first entering of the knife; which accident may happen, if the anterior chamber is small, and the iris is close to the cornea. In order to avoid this error, modern authors (I believe, without any exception,) recommend that the knife should be entered in the direction of the iris, as if the point were to be carried directly against it, but that, as soon as the cornea is penetrated, and the point is in the anterior chamber, the handle of the knife is to undergo a sort of almost imperceptible depression towards the temple, by means of which the blade is to be placed with its flat surfaces parallel to the iris, across the anterior chamber; and it is said that the more quickly this is done, the less chance is there of the escape of the aqueous humour. To all this I object in the most decided manner; the gentlemen who think they do as they say, are, I believe, mistaken; and when they do, they do nothing but mischief. The very turning, or attempting to turn the knife, generally leads to the escape of the aqueous humour—the very thing they want to preserve, and to all the evils it is most desirable to avoid. The knife should, in my opinion, be held flat, and the point is to be introduced steadily in the same direction at the proper place; and, if the operator can neither see nor feel when it has penetrated the cornea, and is in the anterior chamber, the sooner he abandons operating the better. If a man has eyes, he can see when the point of the knife has entered the anterior chamber; and if he has fingers, he will feel when the resistance offered by the cornea is overcome. The young surgeon, instead of practising these manœuvres, which are worse than useless, should practise on sheeps' eyes, until he has acquired that tact which will enable him to know when his knife is in the right place.—*Guthrie on Extraction of the Cataract.*

[To this it may be objected, however, that it is not quite so easy to know by the sight whether the knife has the whole cornea before it, or the whole cornea minus one extremely thin lamina; nor will the absence of resistance always satisfy the young beginner, as the knife will still be pressed by the uncut part of the cornea.—[Ed. *Med. Quart. Rev.*]

CASES OF THE ADMISSION OF AIR INTO THE VEINS.

Case i. Mr. William Burrill, of Salem, aged sixty, was admitted into the Massachusetts General Hospital, on the 16th October 1830. He had a cancerous affection of the left side of the face and neck, of the extent of three or four inches diameter. It was hard at the edges, of a livid red colour, ulcerated in the centre, very offensive, very painful, and had made an impression on the general health. The parotid gland, the submaxillary, the sublingual, and all the textures, excepting the bone, were involved in the complaint. The lower jaw was thought to be diseased, at first, but it afterwards appeared not to be so. In so bad a state of things, there seemed to be little hope of eradicating the disease, and the operation would not have been attempted, had not the patient solicited it.

Considering the extent of the disease; that important blood-vessels would be divided, namely, the facial and sublingual arteries, probably the temporal, and possibly the external carotid; it was thought best to secure the carotid trunk. An incision, for this purpose, was begun opposite the thyroid cartilage, and carried two inches downwards. The platysma muscle was divided, the edge of the mastoid exposed and dissected. Thus far, only a few drops of blood were discharged. The face of the sheath of the great vessels was a little uncovered, when a small effusion of venous blood appeared under the knife, and checked the operation. At that instant a very distinct sound was heard, like the passage of air through water. A few bubbles were seen in the venous blood, the flow of which was immediately arrested by applying a finger on the part. The patient exclaimed, "I am faint." On regarding his countenance, it was not pale, but livid, almost black, and the muscles agitated by a convulsive motion. The respiration became deep, laboured, and stertorous, like that of apoplexy. The pulse being examined at the wrist, was found distinct, but very slow. The wound not bleeding, and very little blood having been lost, the temporal artery was opened, and the blood flowed from it with great freedom. As it flowed, the respiration became more frequent and less laborious, the pulse at the wrist more natural. The leaden colour in the cheeks assumed a reddish tinge, and the alarming character of the symptoms was evidently diminished. About twenty minutes elapsed during these changes. At the end of half an hour, it was thought safe to remove the patient to his bed, where he lay in a state of insensibility for two hours, at the expiration of which he awaked as from sleep, still breathing like an apoplectic. The night was passed without any accident, and on the following morning he was as well as usual, with the exception of a moderate soreness over the thorax, and a headach.

In seven days after the accident described above, the operation was performed without tying the carotid artery.

The diseased parts were surrounded in an elliptical incision, extending from the lobe of the ear to the upper part of the neck, and including the submaxillary, the sublingual and parotid glands, all of them in a morbid and disorganized state, and they were all entirely removed. The hæmorrhage was copious, but readily arrested, with the exception of that from a large vein, which, from its depth, under the jaw, could not be distinguished so as to admit the application of a ligature, and was therefore compressed by a sponge. The veins below the wound were compressed during this operation. The patient experienced a slight faintness, which soon passed off. He had no bad symptoms; and on the 10th of December, the wound being nearly healed, he requested his discharge, which was granted.

Case ii. Nancy Bunker, of Trenton, of Maine, married, her age thirty-three. Three years since, she noticed a hardness in the right breast, which increased till it involved the whole gland in a tumour, very hard, moveable, yet connected with the pectoral muscle by a morbid adhesion. The nipple is drawn in. The axilla is occupied by a considerable tumour, of a globular form, and quite hard. An operation was performed on the 24th December, 1831.

The patient sat in a chair. The right arm was extended, raised above a horizontal line, in order to give tension to the skin, and permit access to the arm-pit, and was supported in this position by an assistant. The skin on the surface of the breast, with the diseased nipple, were included in an oval incision; the breast was dissected from the pectoral muscle, and left connected with the axillary glands while the extirpation of these glands was effected. As they adhered to the great axillary vessels, they were cautiously detached by dissection, and by insinuating the finger, where the cellular substance was loose, between the tumour and the great vein. This separation was nearly effected, only a slight connexion still existing at either extremity of the tumour. Proceeding to separate it, at the outer part of the axilla, a vein was divided, and a small quantity of venous blood discharged. Scarcely was this done, when the patient struggled, her complexion changed to a livid colour, and at the same instant the bubbling or clucking noise, which had not been noticed before, was heard, though indistinctly; but the place from which it issued was not visible, the surrounding skin and fat lying over it. On this, the axilla was immediately compressed. The patient became insensible, breathing as in apoplexy. The tumour was at once separated. The posture of the patient was changed, and she was supported by those around. Some brandy was poured down the throat, and ammonia introduced into the nostrils. The pulse, however, became less distinct every instant. Cloths dipped in hot water were thrown over the extremities. Strong frictions were applied to the chest, and to all parts of the body. Considerable quantities of brandy were again poured down the throat. At this moment the livid colour of the cheeks gave place to a suffusion of vermilion red, and no glow in the cheek of a youthful beauty ever gave one so much pleasure as that flush. But the flush soon passed off; the lividness reappeared; the respiration became more feeble; pulse at the wrist scarcely perceptible; and, notwithstanding the redoubled applications of external heat and moisture, the extremities and the whole body cooled rapidly, and presently the respiration ceased.

As a last effort, the larynx was opened, and the inflation of the lungs by a bellows was put in operation in a speedy and perfect manner, imitating the movements of inspiration and expiration with great exactness, continuing at the same time the general application of heat and frictions to the whole surface. These measures were employed for about twenty minutes longer. At the end of this time, there was no remaining hope of the restoration of the patient to life. The friends being anxious to take advantage of a vessel then sailing for their home, the body was soon after removed, and no opportunity afforded for examination.—*Dr. Warren, in the Cyclopædia of Practical Medicine and Surgery, a Digest of Medical Literature, edited by Dr. Hays.*

[This is an American publication. It bears the marks of great industry, and will be very useful when finished. It is expected that it will be completed in forty parts, and a part will be published every month, if practicable. Part I. is dated July 1833, Part II. October 1833, and Part III. January 1834. The whole will make eight large volumes.—*Ed. Med. Quart. Rev.*]

### SEPTAN AGUE?

Robert Kidson, æt. sixty-two, a mason, presented himself to Dr. Duncan on the 29th April, with asthma, of long standing, but which had been aggravated within the few preceding weeks. On each of the three preceding Mondays, he had had an attack of shivering, followed by heat. On questioning him, it appeared, that forty years ago, while residing in the neighbourhood of Northallerton, he had an attack of tertian ague, subsiding into quartan. He has not been out of Liverpool for the last twenty years.

The treatment was directed to the relief of the asthma, and to the improvement of the general health. No remedies were used specifically against the ague.

The shivering returned, as usual, on the two following Mondays; but his digestive system is now in better order, the chest symptoms are mitigated, and on Monday last (19th May), he escaped without his expected attack.

Was this an attempt at the renewal of morbid action, the latent poison taking advantage of the enfeebled state of the constitution, after the lapse of forty years? There are instances on record, we believe, of intermittents recurring after intervals nearly as long. Two or three years ago, a patient presented himself at the Dispensary, with ague. He was an old soldier, who, while in the Walcheren expedition of 1809, had, in common with most of his comrades, suffered from intermittent fever. During the interval, of more than twenty years, he had no symptoms of the disease. His last attack he attributed to exposure to a cold east wind.—*The Liverpool Med. Journal, June.*

### CASE OF A WOUND OF THE TRACHEA AND ŒSOPHAGUS, IN WHICH THE HEMORRHAGE STOPPED WITHOUT THE ASSISTANCE OF ART. BY DR. A. NEUMANN.

A joiner, of the name of F—, attempted to kill himself on the 9th of June, 1830, at four o'clock in the morning, by cutting his throat with a sharp razor, after having shaved himself. When his wife, who slept in the same room with him, awoke, an hour afterwards, she saw her husband, covered with blood, sitting at a table, and writing with great earnestness. The author was quickly called in, and found the following injuries.

There was a cut, about three inches long, in the superior and anterior part of the neck, immediately under the submaxillary and sublingual glands, which, when the head was bent backwards, showed a truly horrible gaping wound. The sterno-hyoideus and sterno-thyreoidei of both sides, and the omohyoideus of the right side, were found to be completely cut through, and only a few fibres of the left omohyoideus remained entire. Besides this, the ligamentum thyreoideum medium, with the mucous membrane of the whole larynx on the anterior and posterior sides, together with the superior cornua of the thyreoid cartilages, with their ligaments; and, lastly, the anterior and lateral parts of the œsophagus, were so completely cut through, that a finger, introduced to examine, could with facility reach that surface of the mucous membrane of the œsophagus which is attached to the spine. The trachea and œsophagus, therefore, with all the anterior parts, were entirely cut through, as far as the posterior wall of the œsophagus, (which could hardly be divided without injuring the spine,) and still no fatal hemorrhage took place, although the man, as he afterwards said himself, had done nothing to stop the bleeding, except that, after washing his neck and hands with cold water, he put a dry woollen cloth on the wound, and fastened it round his neck. Yet the author did not find it necessary to take up any artery, nor even afterwards, during the process of suppuration, on account of any secondary hemorrhage. It is true, however, that, according to the above description of the wound, it would not have been easy to have hit upon a place in the trachea and œsophagus where there are less vessels of consequence than directly in this spot. But our surprise must be so much the greater, that the parts adjacent to the trachea and œsophagus could remain uninjured, the depth of the wound being so very great. It is probable that the cut was so directed forwards on the middle of the windpipe with the right hand, that the point of the razor was pushed through the trachea into the œsophagus from the left side, and then immediately drawn out again anteriorly. By this means the wound must have had a very extraordinary depth in comparison with its breadth, since the blunt end of the razor only pushed back the important organs on each side, without injuring them.

The strength of the patient, after the incision, which enabled him to sit up, and even write for hours afterwards, showed how few vessels had been divided, and how little blood had been lost.

In a physiological point of view, it was remarkable that, although the larynx itself had remained whole, still the patient was not able to speak, till a thick wet cloth was applied to the wound, when it was found that his voice had lost little or nothing of its clearness.

The patient lived till the 22d June, full fourteen days, and died at last from weakness, partly from his age, (for he was fifty-five,) and partly from delirium tremens, produced by drinking: indeed, it was in a paroxysm of this disease that he committed the deed. Under such circumstances it was not to be expected that the edges of the wound would diminish in extent, although the bloody suture, as well as Köhler's cap, were applied for that purpose; and the patient's surviving a fortnight is the more surprising, as the greater part even of the fluid nourishment injected flowed out again through the external wound. This may show, however, that such a wound need not be accounted absolutely mortal, either on account of the bleeding, or of the suppuration, should it occur in a young and healthy person.—*Gräfe und Walther's Journal.*

### *Practical Observations on the* Method *of* Reducing Dislocations *of the* Humerus *downwards by the Heel in the Axilla.* By G. Paolo Cumano, Professor of Surgery at Trieste.

This method of reduction was known to Hippocrates. It has been mentioned in the writings of almost every author since his time, being praised by some and depreciated by others, and frequently confounded with some twelve or fifteen other methods prescribed by the ancients for reducing this dislocation. In our own times it has received the preference from Sir Astley Cooper, at least in recent cases.

Professor Cumano relates eight cases in which it has succeeded. The first, a young athletic sailor; the second, another sailor, æt. eighteen; the third, a peasant, æt. forty; the fourth, a sailor, æt. twenty-seven; the fifth, a man, æt. fifty-three; the sixth, a man, æt. fifty; the seventh, a woman, æt. fifty; the eighth, a man, age unknown. In the first six cases the dislocation was downwards. The first, which was the most recent, had existed only an hour; the last, which was of the longest standing, four days. We have mentioned the ages, in order to fix the period at which this dislocation may occur; and, after particular research upon the subject, we are inclined to believe that, like that of the femur, it is rarely met with after sixty years of age.

The two other cases deserve more lengthened notice.

Case i. In the evening of the 14th of July, Anna Vida, æt. fifty, a very stout woman, while drawing some water, suddenly fell down. Being called to her the following morning, says the author, I found her on the bed, suffering severely from a dislocation of the left humerus, with various bruises, and a lacerated wound on the summit of the joint. The forearm was bent, and the arm raised and removed from the trunk, so that the patient's hand was in contact with her head. The dislocation, which had existed twelve hours, was downwards and forwards under the pectoral muscle, and was attended by severe pain down the arm, and a sense of distension in the fingers. The reduction was accomplished by the heel in the axilla. On the following day the arm was discoloured by ecchymosis along the biceps muscle, particularly at its lower part, which was swoln and painful. The application of a few leeches restored the mobility of the arm, excepting in the direction upwards, the deltoid muscle being partially paralytic; but in the course of a year this symptom also was removed under the use of friction.

In one of the preceding cases, where the dislocation downwards had existed four days, perfect motion of the limb was not regained till after two months, during the whole of which time there was a sense of distension in the fingers. The author attributes the paralysis to the length of time that the head of the bone was pressing upon the brachial plexus of nerves, and to rheumatism, from which the patient had formerly suffered. In the case of

Anna Vida, he believes it to have been dependent on the internal disease, which occasioned the loss of consciousness at the time of the fall.

Case ii. Lazzaro Marpurgo fell from a carriage on the 4th of October, 1833, in the neighbourhood of Gorizia; the humerus was dislocated, but the nature of the accident was not discovered till the patient arrived at Trieste, twenty days afterwards. Dr. Forté and Professor Cumano soon recognised a dislocation forwards. The patient was bled twice largely, and on the twenty-fourth day the head of the bone was returned to its socket on the second trial with the heel in the axilla. Half an hour after the reduction the patient had a rigor, attended with severe pain down the arm, which was followed by general heat and sweating, accompanied by alleviation of the pain. In the course of a fortnight the arm had regained all its motions excepting those of the deltoid, which was affected by a partial paralysis, consequent on the pressure of the brachial plexus and the circumflex nerve: this, however, gradually disappeared.

The author has several times since had the opportunity of employing this method with success, as also have his colleagues, (excepting in a few cases,) whenever the dislocation had existed only a few days, or where the head of the bone was very forward, or in those cases in which unsuccessful attempts had been previously made at reduction. In such cases the author employed indifferently the method of Desault, the pulleys of Cooper, or extension upward, in the manner recommended by Mothe. This conclusion well displays the uncertainty of our knowledge on the subject; for Desault recommended that the extension should be made downwards, Cooper prefers it outwards, and Mothe directly upwards; and yet the author finds them equally successful. This arises from ignorance of the nature of the injury, and of the exact position of the head of the bone, each author having deemed success a sufficient apology for his method. Again, out of eight dislocations, three were followed by paralysis; but it may be questioned how far this depended on the method employed in the reduction.—*Gazette Médicale.*

[It is not difficult to estimate the real value of the method of reduction by the heel in the axilla. There are two principles on which these and all dislocations may be reduced: one, on that of direct extension in such a direction as will meet with least opposition from muscular contraction; the other, the employment of a lever, by which one hand may acquire the power of many. The former has the inconvenience of requiring assistants, who, from want of skill, may embarrass the operation. The latter is entirely in the power of the surgeon; but, as a great portion of the force must fall upon the affected part, injury may be done to the nervous trunks in the vicinity, and paralysis may ensue. By care the objections to the former may be overruled, and, in our opinion, it should therefore be preferred whenever the requisite assistance can be obtained: where, however, this cannot be done, the surgeon must endeavour to employ the latter, provided always that his dorsal muscles are equal to the task.—*Z.*]

TREATMENT OF ANEURISM.

We cannot terminate the present article without noticing a table attached to M. Lisfranc's thesis (our analysis of which we have now completed), which at once shows immense literary research, and may serve as a foundation for several curious and useful deductions.

The table contains 242 cases of aneurism, and indicates, in parallel columns, the work in which the case is described; the surgeon treating; patient's name, age; artery affected, and species of aneurism; process employed; accidents; date of the ligature coming away; and termination. It is subdivided into sections, according to the different methods employed.

I. II. and III. contain eighteen cases which were treated by refrigerants and Valsalva's method. In five cases treated by Valsalva's process there are four cures (carotid and subclavian aneurisms); the remaining thirteen, treated by styptics, refrigerants, &c., give six failures, six cures, one uncertain.

IV. Compression between tumour and heart, thirteen cases; eight unsuccessful, five cured.

V. Compression on the tumour, five cases; one cure (aneurism by anastomosis of temporal artery).

VI. Compression on the whole limb, five cases; three cures, two failures.

VII. Compression without indication of the spot, twenty-four cases; cures eleven, failures thirteen.

VIII. Ligature after the old method, thirty-one cases; failures six, cures twenty-three, two doubtful.

IX. Anel's, or Hunter's method, 151 cases; failures forty-five, cures 106.

X. Brasdor's method, fourteen cases; four cures, ten failures.

XI. Torsion; *refoulement*, one case; cured without accident.—*Lancet.*

MALPOSITION OF THE HEART.

Andrew Dunlevie, æt. fifty-three, is at present attending Dr. Duncan, with chronic bronchitis and asthma, but without any signs of effusion into the chest. The heart beats on the right side, the chief impulse being felt between the fourth and fifth ribs, immediately below the nipple. The pulse is regular, and of about equal strength in both arms; the impulse on the right side of the chest is natural, and none is felt on the left side. He was for thirteen years in the army, and served in the Peninsula, and in Canada, but has always enjoyed good health until lately. He does not remember that he ever felt pulsation on the left side.—*The Liverpool Med. Journal, June.*

ADDITIONAL CASES OF CLUB-FOOT TREATED BY DIVISION OF THE TENDO-ACHILILS.

Henry Lezing, ætat. seven, the son of a gardener, in the neighbourhood of Hanover, was born with both his feet deformed. By constant surgical treatment their proper form was partially restored, especially that of the left foot. When he came under Dr. Stromeyer's care, the right limb was much wasted, the edge of the foot turned inwards, and the toes downwards. When he attempted to walk, he rested but slightly on that side, the outer edge of the foot being in contact with the ground, and the toes were directed very much inwards. Its voluntary motion was extremely limited, but it might be returned to its natural position without much force. As time was an object with the patient, the division of the tendo-Achillis was proposed, in order to equalize the action of the flexor and extensor muscles. The operation was performed on the 26th of August, 1832, in the same manner as in the two preceding cases. The external wounds were united, and the cut edges of the tendon were adhering on the fifth day.

Although it was foreseen that the union of the tendon would be more firm in so young a person than in an adult, yet extension could not be attempted till the eighth day, on account of the tenderness of the wounded part. The foot was readily restored to its natural position, under extension gradually increased for fifteen days; and it then formed an angle of about seventy degrees with the anterior surface of the leg. It was left in this position during three weeks. On removing the apparatus, I found, says Dr. Stromeyer, the intermediate substance thinner than the tendon, but not above two or three lines in length. The form of the foot was restored for the time; but in a few hours the bad position returned, in spite of the ordinary instruments which were applied, but removed again in a short time on account of the pain which they occasioned; so that the boy's state was not in the least ameliorated.

The error in this case was, that extension was not commenced sufficiently early to lengthen out the substance uniting the tendon. A second operation was proposed, in order that this fault might be corrected, but the parents of the boy rejected the proposition.

Case ii. Henry Linse, ætat. thirteen, the son of a peasant, of Schulenburg, near Hanover, had been affected with a club-foot since the age of four, without any known cause. As nothing had been attempted for its relief, it had continued to get gradually worse. He came under Dr. Stromeyer's care in August, 1833. The deformity was greater in this case than in that of Lezing: he walked entirely on the outer edge of the right foot, and this occasioned so much pain, that the poor boy was forced to give up every kind of motion. The toes, more especially the great toe, were drawn inwards by the action of the flexor longus pollicis pedis, the tendon of which could be seen in a state of extreme tension. The outer edge of the foot was rendered very hard by constantly supporting the weight of the body; the leg was wasted, and the foot scarcely moveable; considerable force, however, would partially restore it to its natural position.

Before dividing the tendo-Achillis, Dr. Stromeyer cut out that of the flexor longus pollicis pedis, introducing the knife underneath the tendon from the inside of the foot, about half way between the toe and the heel. On the third day an extensile apparatus was applied; and on the eighth a great improvement was perceptible in the direction of the foot. The tendo-Achillis was then divided on the 15th of August. On the fifth day there was adhesion of the tendon, and extension was applied; and, on the tenth day, the foot formed an angle of seventy degrees with the thigh.

In four weeks' time the apparatus was removed, and the foot placed in an iron boot; and extension was kept up during the night, by means of a screw. The boy afterwards began to walk, and that with so much ease, that he was permitted, on the third day, to walk out in a well-paved street, for a quarter of an hour. He transgressed, however, the directions of the doctor, and remained out three hours, with no inconvenience, saving fatigue. A fortnight after, he was in a state to walk four or five leagues. The foot had then recovered its natural form, excepting a slight inclination inwards; it was perfectly moveable, and had increased in size. Six months afterwards he was in the same state as to the foot, but the leg had continued to enlarge.

Case III. Ferdinand Wesl, ætat. nine, a native of Celle, was born with a club-foot on the right side. By surgical treatment the state of the foot was somewhat ameliorated during the early years of his life. When he was five years old, he came under Dr. Stromeyer's care, who applied the ordinary instruments, the iron boot, &c., with which the boy was able to walk. This plan was continued for five months. He remained in the same state for three years, when, being attacked with illness, his foot became worse. In December, 1833, Dr. Stromeyer again saw him. The state of the foot was then much worse, walking being nearly impracticable, on account of the pain which it occasioned. The dorsum formed a convexity with the anterior surface of the leg; the toes were turned inwards, and drawn towards the heel, and the great toe was bent backwards in a very singular manner.

January 10th, 1834. Division of tendo-Achillis.

On the 15th, adhesion of the cut edges and extension commenced. The foot was readily straightened.

It was left in the apparatus till the twenty-eighth day after the operation, and then put into the boot. The child walked immediately with the greatest ease.

As the point of the foot was still directed a little inwards, the tendons of the flexor longus pollicis pedis was divided, and within three days afterwards that of the extensor. The foot was placed again in the apparatus, and the great toe was drawn forwards. In eight days' time he was allowed to use the boot. The division of the tendons of the muscles of the great toe, so far from paralysing its motions, appeared rather to have allowed them to be more free. By the end of March the foot was restored to its natural form and action.

Case iv. Mlle. Brandes, ætat. nineteen, a native of Hanover, having suffered much in her childhood from scrofulous disease, was seized with paralysis at the age of two years, while running across the room. She however recovered from the paralysis, excepting that it left great weakness of the right leg, the foot of which became gradually contracted. Every attempt at restoring the foot was not only useless, but attended with considerable pain.

When this young lady came under Dr. Stromeyer's care, the dorsum of the foot formed a straight line with the front of the leg, in consequence of the projection of the astragalus; the foot was turned slightly inwards, so that the point of support during walking was the external surface of the metatarsal bone of the little toe. With a cork heel nearly four inches high, the sole of the foot scarcely touched the bottom of the boot; the leg was wasted, and she had no power over the motions of the foot. External force had but little influence in removing the deformity.

March 11th, 1834. Division of the tendo-Achillis. On the fifth day there was union of the cut surfaces, and extension was commenced. In three weeks the foot formed an angle of seventy degrees with the leg; a boot was then applied, which kept the leg in the same position. This occasioned œdema of the foot, which disappeared in a week, under the use of friction with alcohol. The young lady then began to take regular exercise, but walked with some difficulty at first, in consequence of her habit of using the limb in a perfectly different manner. Nevertheless, as she constantly exercised it, her progress was rapid. Three weeks after her first attempt she walked firmly without any support, and the form of the foot was restored to its natural state.

In these last three cases it is impossible to describe the length of the intermediate substance, as it requires a most diligent examination to discover one part of the tendon thinner than another. When the position of the calf is remembered, it will be seen that it must be of considerable length. The two boys, with casts in plaster of their feet in the original state, have been seen by the Society of Medicine at Hanover. Dr. Stromeyer, in publishing these cases, has endeavoured to direct the attention of the profession to an operation, which, though extremely useful, has of late fallen into disuse.—*Archives Générales, Juin.*

### PARALYSIS OF THE PORTIO DURA.

Two cases of this interesting local paralysis have lately occurred in the practice of Dr. Carson, jun. The first, in a middle-aged man of rather full habit, came on suddenly during a severe coryza, consequent on exposure to cold. The other was the sole remains of a complete hemiplegia of the left side, which occurred thirty years ago. In neither of them was there observed that absence of distortion mentioned by Bell, during the quiescence of the muscles of the face; the mouth was continually drawn to the sound side. In both cases, at first, the tongue appeared to be thrust out to one side, but this appearance altogether arose from the distortion of the mouth. On comparing it with the teeth, it was evidently protruded straight forward. One of the most remarkable symptoms was, the total absence of nictitation in the eyelid of the affected side. This, in the case of long standing, has been followed by chronic conjunctivis, with destruction of vision. The inflammation of the eye came on about seven years ago, when he went to work in a sal ammoniac manufactory. No doubt, the eye not being occasionally lubricated by the lid, the acrid vapours of the ammoniac had excited such a degree of irritation as to destroy vision. There is in this case a degree of deafness in the ear of the affected side, such as that, though he can perceive sounds, he cannot distinguish them. Query, can this be attributable to a paralyis of the muscles which tighten the tympanum, and move the small bones of the internal ear; and if so, may not this deafness be a distinguishing symptom between the affection arising from disease of the brain, and the rheumatic affection arising from cold?

By the repeated application of blisters behind the ear, leeches to the seat of the origin of the nerve on the face, and the use of calomel and jalap purges so as to affect the mouth, the patient, in the course of a month, completely recovered.—*The Liverpool Medical Journal.*

### CRYING OF THE FŒTUS IN UTERO.

I was some time since called to the wife of a blacksmith at Preston, about two miles from my residence, who was in labour with her tenth child. I had attended her in several former confinements, and she had always had quick deliveries, as the pelvis was unusually capacious, and her pains were active. After I had been a few minutes in the room, I proposed and made an examination, and found the face presenting, and making its descent into the pelvis, the chin resting on the os pubis.

A few strong pains succeeded, and I again examined, to ascertain if the face had made any advance. I found it had done so, and that it was pressing on the perineum; but, in making this examination, my finger passed freely into the mouth of the child, and it immediately gave a convulsive sob, and cried aloud, to the great terror of the mother and of the bystanders, when they found that it was still in the womb. I had great difficulty in calming the agitation produced by this event upon the woman, whose pains were suspended for nearly an hour, but I eventually succeeded by explaining that the face was presenting, and that, from the circumstance of my having passed my finger into the mouth, the air had gained admission, and enabled the child to breathe; this, with a little spirit and water, and a dose of the ergot of rye, succeeded in bringing on the uterine action, and, after two pains, the child was expelled alive and well, at least one hour after it had respired and cried in the womb. I am not aware that any instance similar to the above is on record, but I consider it one of consequence as a physiological and medico-legal point, and one which will throw additional difficulties in the way of that clear proof which is so desirable in cases of infanticide.—*Mr. Tomkins, in Lancet.*

### TREATMENT OF DIARRHŒA IN INFANTS.

In treating all cases of purging in infants, our attention should be especially directed to two objects: first, the feel and appearance of the abdomen; secondly, the character and kind of the evacuations. Upon feeling the abdomen, (which should be done when the child is in an easy posture, and not crying,) if it be found full, hard, or irregular in its shape, a purgative will almost certainly be required, notwithstanding the frequent evacuation of fecal matters. The most effectual purgative in such cases is a grain or two of calomel, either combined with jalap or scammony, or rhubarb, or given by itself, and followed in a hour or two by a dose of castor-oil, or solution of salts in almond emulsion. This purgative is to be repeated according to circumstances, and in the meantime a cretaceous mixture may be given at intervals, or, what is often preferable, a solution of carbonate of soda or potassa, in mucilage or emulsion.

If the evacuations be hard, lumpy, scyballous, or pasty, or of a green, or leaden, or blackish colour, the same plan is required; and the aperients must be continued daily, or as often as may be found necessary, till the evacuations become of a better consistence and better colour.

When the use of the more active purgatives shall have relieved the complaint, the cure must be perfected by giving a small portion of ipecacuanha, a grain or two of rhubarb, and a few grains of chalk, with the addition of a little aromatic, twice or thrice a day.

But, when the abdomen is not fully hard, or irregular in shape and feel, but flabby and collapsed; and when the stools are thin and watery, pale coloured, or white and frothy like yeast, the use of calomel and purgatives is to be rejected, and other more appropriate means must be employed. The case now requires the use of cretaceous medicines, kino, and catechu; opiates must be occasionally interposed, and an attempt must be made to improve the secretions, by three or four grains of Hydrarg. c. Creta, or a grain of Pil. Hydrarg. rubbed into a powder with four or five grains of carbonate of magnesia: sometimes a combination of ipecacuanha, rhubarb, and soda, very effectually answers this purpose. Change of diet, as Dr. Underwood recommends, is indispensable; and change of air is sometimes to be recommended.—*Dr. Merriman's Notes on Underwood.*

CASE *of* EXTIRPATION *of a* TUMOUR *of the* NECK, *in which the Carotid Artery and Internal Jugular Vein were tied: with Remarks.* By WILLIAM GIBSON, M.D. Professor of Surgery in the University of Pennsylvania.

George Washington Reynolds, seventeen years of age, came to Philadelphia from Delaware, in November, 1832, and placed himself under care of Dr. Horner, on account of a tumour of the size and shape of a cocoa-nut, which occupied the whole of the left side of the neck. Dr. Horner referred him to me, and at the same time requested the opinion of Dr. Physick on the case. The friends of the patient stated that the swelling had made its appearance five years before, that it arose without evident cause, and had gradually increased to its present magnitude. The boy now sought relief, on account of the difficulty of breathing and of deglutition he experienced, and which increased with the growth of the tumour. In other respects he felt no inconvenience from it; his complexion was florid and healthy, and his constitution apparently sound and vigorous. In consultation, it was determined that nothing less than extirpation would afford a chance of recovery, and I was requested to undertake the operation. Previously, however, I thought it advisable to reduce the patient by bloodletting, low diet, and other antiphlogistic means, both to diminish hæmorrhage, and to guard against inflammation. Having fulfilled these indications, I commenced the operation, (November 20th,) assisted by Drs. Horner and J. R. Barton, in the theatre of the Alms-house Infirmary, in presence of several hundred students. The patient was placed at full length on a narrow table, his head inclined to the right side and supported by a pillow. An incision two inches long was made over the course of the carotid, low in the neck, and that vessel tied by a single ligature. Over the most prominent part of the tumour, commencing immediately under the angle of the lower jaw, and extending nearly seven inches, another incision was made through the integuments; continuing the line thus chalked out, layer after layer of condensed cellular membrane, of fasciæ, and the fibres of the platysma myoides, were successively divided, as well as those of the sterno-mastoideus, which last muscle was spread out by pressure of the tumour, and converted into a thin muscular expansion, intimately incorporated with the platysma, and rendering it difficult to distinguish one from the other. During this stage of the dissection the internal jugular vein was exposed, tied by two ligatures, and divided between them. The ends of the vein were then dissected from the surface of the tumour, and turned to one side. In order to get round the tumour, and raise it from the cavity in which it was deeply imbedded, it became necessary to separate the integuments, fasciæ, platysma, and other coverings. This proved very difficult, and was not accomplished without great risk, owing to the distribution of the par vagum and descendens noni nerves, both of which lay on the surface of the tumour, and were closely attached to it. I soon found it was impossible to get out the tumour, and at the same time preserve the descendens noni. I therefore cut it across. Instantly a slight shudder passed over the patient's frame; but the effect was momentary. I determined, however, not to divide the par vagum, dreading the result either immediately or remotely. I was obliged, therefore, to dissect along the edge of the nerve for five inches, and succeeded in detaching it from the tumour, to which it had formed a very close adhesion. This was the most painful and difficult part of the operation, and nothing but the uncommon composure and fortitude of the boy, perhaps, enabled me to accomplish my purpose, for he remained during the whole operation motionless, and neither complained, sighed, nor groaned. The par vagum having been thus pushed aside and out of danger, I continued the dissection, taking up occasionally small vessels, sometimes separating with the handle of the knife the adhesions between the tumour and a firm fibrous sac, in which I now found it enclosed, at other times using the edge of the knife until I reached the base of the tumour, which was intimately connected with the pharynx and œsophagus, and to remove it from which required every possible precaution. In this also I at last succeeded, though not without the division of four or five vessels, which at first shed blood freely, but were soon tied, or shrunk of their own accord. The action of the pharynx and œsophagus was distinctly seen, even at a distance, whenever the boy imitated the action of swallowing, or took fluid by the mouth. The cavity left by the tumour was even larger than had been anticipated, owing to the influence of pressure upon all the surrounding parts. To guard against return of hæmorrhage, the patient was left on the table for twenty minutes, and the edges of the wound held together temporarily. No hæmorrhage occurring, the wound was dressed regularly by adhesive straps, &c. and the patient put to bed. The operation lasted thirty-four minutes.

The tumour having been cut open and examined in presence of the class, was found to consist of a medullary-like matter, of rather firmer texture, however, than that usually met with in fungus hæmatodes. The idea was at once impressed upon my mind, and for the first time, that such indeed was the nature of the disease. The unusually healthy appearance of the boy, and in particular his florid complexion, (circumstances so uncommon in fungus hæmatodes, which is almost invariably accompanied by a sallow, cadaverous countenance,) had prevented any of us from entertaining suspicion of the kind. There was not, moreover, the elastic feel, and deceptive sensation of fluctuation, so characteristic of fungus hæmatodes.

[The patient was discharged cured in six weeks after the operation; but the disease soon returned; and, as it was then obviously a case of fungus hæmatodes, nothing could be done, and the patient died soon afterwards. The author concludes with the following remarks. Ed.]

There are two points connected with the operation just detailed, which give it an interest it might not in other respects, perhaps, be entitled to,—the application of a ligature to the internal jugular vein, and the division of important nerves. It is an opinion generally received among surgeons, that large veins cannot be tied without great risk of inflammation of their internal surface speedily following, and proving fatal by extending to the heart. Such certainly has been the result in numerous instances in European practice, but it is equally certain that the occurrence in this country is extremely unusual; for, out of a great many instances in which I myself have tied varicose saphenæ veins, and have seen the operation performed by others, I have never met with a single case of injury, much less of death, from such a cause. The only instance, indeed, that has ever come to my knowledge of death from tying the saphena vein, occurred a few years ago in this city, in the practice of a respectable surgeon. There were circumstances, moreover, connected with that case, which rendered it very doubtful whether the unfavorable issue was owing to the operation, or to other causes. I think it very probable, therefore, that the constitutions of patients in this country, (owing to all classes of people being well fed and clothed, and little exposed to hardships,) are generally superior to those of Europeans, and, as such, more capable of resisting the operations of injury or disease. Whether this be true or not, however, it is certain, judging from the details published by European writers, that the patients that have fallen a sacrifice to phlebitis, occasioned by the ligature of veins, have very generally been among the lowest classes, whose constitutions were of the worst kind, and whose operations were performed in the crowded hospitals of large and unwholesome towns. I do not wish to be understood, however, to say that there is no danger from including a large vein in a ligature. On the contrary, I am well persuaded that there is always more or less risk, much greater risk indeed than would follow the tying of a large artery. I only mean to imply that there is less danger in tying a vein than is commonly imagined. Influenced by this opinion, I ventured, in the case I have related above, to tie the internal jugular in two places. There are only a few examples on record, I believe, in which this vein has been tied, and most of them had a successful termination. The operation was first performed, there is reason to believe, by Dr. Simpson, of St. Andrew's, in Scotland, eighty years ago, and the patient recovered without a bad symptom. Mr. Simmons, of Manchester, in England, also tied up the internal jugular, and with a similar result. Giraud has recorded a case, in which a French surgeon, at Toulouse, tied the trunks of the common carotid artery and internal jugular for a wound from a musket-ball. The patient had no unfavorable symptoms as far as the sixth day; but it is not stated whether the man recovered. In this country the internal jugular has been tied by Drs. Mott and Stevens, of New York, by the former in 1828, and by the latter in 1830. Both patients recovered. Some interesting remarks on the ligatures of veins, in which cases and experiments have been detailed, have been published by Trousseau, and may be found in the 14th vol. of the "Archives Génerales de Médécine." The author is induced to conclude that there is less danger from tying a vein than is commonly imagined, and that the danger is often owing to prematurely pulling the ligatures away, and other mismanagement on the part of the surgeon. Fatal cases of wounds of the jugular veins have been reported, and the death of the patients attributed to the introduction of atmospheric air.

The division of the descendens noni, in the case of Reynolds, produced little or no inconvenience, it will be seen, to the patient, owing no doubt to this nerve being chiefly destined to supply the muscles of the neck, and holding no important connexion with the vital organs. The dissecting up the par vagum, by which it was more or less disturbed, probably gave rise to the erysipelatous inflammation of the abdomen, this nerve having an intimate relation to the stomach, intestines, &c. A division of it during the operation would probably have produced great disturbance in the animal economy, or have led, remotely, to the patient's death. The only further remark I deem it necessary to make in relation to this case, is, that could I have known the tumour to have been of the nature of fungus hæmatodes, I should certainly not have undertaken to remove it, upon the ground, that there is not a single well-attested case on record in which this inveterate malady has been successfully removed by extirpation, and very few where the patient has recovered after amputation.---*American Journal of Medical Sciences.*

### A CASE OF OSSIFICATION OF THE MUSCULAR TISSUE.

By David L. Rogers, m.d., Lecturer on Surgery in New York.

In June, 1832, Dr. R. was first consulted in the case of James Mulwill, aged thirteen years. His father stated that his son, from his infancy, had been in good health, and was remarkable for animation and a high flow of spirits. About six months ago, it was perceived that his health began gradually to fail, and without any perceptible cause. At first a loss of motion in the arms was noticed: he was unable to raise them to his head, or carry the elbows to any great distance from the body. The motion of the right arm lessened every day, until it was permanently fixed to the side of the body. Shortly after his head inclined forwards and downwards on the sternum. At the time Dr. R. first saw him, his appetite and digestion did not seem to be impaired; slept well at night, and the bowels were regular. On examination, it was found that the pectoralis major muscle was ossified at its superior part, and extended in the direction of the clavicle to the arm, the bony deposits forming high and irregular elevations. The sterno-cleido mastoideus was ossified from the sternum to its middle portion, with several elevations. The back exhibited the greatest quantity of ossific matter, having a tubercular appearance. The scapula was fixed to the ribs, and studded with bony excrescences. All the muscles going to the scapula appeared more or less affected, viz. the trapezius, rhomboideus, subscapularis, &c. The latissimus dorsi formed a large bony plate, from its origin to the angle of the scapula: at this part it had united to the ribs, forming a large tubercle. The longissimus dorsi was in a similar condition, extending upwards along the spine, resembling a splint, and to this may be attributed the entire loss of motion in the lumbar vertebræ.

The treatment was various, and may be considered a series of experiments to check the predisposition to the formation of bone.

His general health at this time not being materially affected, recourse was had to alteratives, consisting of the different preparations of mercury with sarsaparilla. Having used these for a length of time without benefit, the acids were employed without effect, viz. the nitric, muriatic, and sulphuric; the carbonate and phosphate of iron were administered with the same result; the iodine was also given, but without advantage.

Finding at the expiration of three months that no change for the better had taken place, and that the bony depositions had increased, all active treatment was now abandoned, and he was directed to live principally on salted provisions: the object was to produce a state of his system resembling that of scurvy, as it is known that bony depositions do not take place in this disease, and that fractures, which have been united for several years, are sometimes separated in the scorbutic diathesis.* Until this time he had been an office patient, but, from some cause unknown, he omitted to call for several months.

In March, 1833, he was visited at his residence: he was much changed; his general health had suffered; had lost his high spirits, and was very irritable; had a diarrhœa; was greatly emaciated; the ossific depositions had, in some respects, changed their situations; the sterno-cleido mastoideus muscle had become free, and the head returned to its erect position; many of the tubercles of the back had been absorbed, and others formed in different places. Bony depositions had taken place also in the muscles about the trochanter major, particularly on the right side. He was compelled to lie in bed, for the least movement produced excruciating pain. A large collection of matter formed in the thigh near the joint, which, when discharged, afforded some relief; but the constant pressure on the bony tubercles on the back caused extensive sloughing, and, after three weeks of great agony, he expired.

The body was partially examined twelve hours after death. The glands of the mesentery were enlarged; no ossific matter in the vascular system, but it seemed to be confined entirely to the muscular tissue. The parts within the abdomen and thorax appeared to be healthy. The pectoralis major and minor muscles were united into one, and attached to the ribs by solid bone. During this part of the dissection a large abscess was opened in each side, contain-

* Lord Anson's Voyage around the World.

ing about six ounces of pus; the tendinous parts of the muscles were not affected. The muscles of the back were all more or less in the same condition. Specimens of the latissimus dorsi, longissimus dorsi, subscapularis, and pectoralis muscles, are preserved in the museum of Dr. Rogers. In several instances spicula of bone projected from the muscles one or two inches; and no doubt, from the irritation they occasioned, abscesses were formed.—*Ibid.*

HYDATIDS OF THE KIDNEYS, PASSED BY THE URETHRA.

Elijah Jones, æt. twenty-seven, a comb-maker, of pale complexion and slender form, applied to Dr. Duncan on the 13th May. He brought with him several portions of a membranous looking substance, having a pearly, semi-opaque, pulpy appearance, and which he said he had passed with his urine three days previously. He stated that he made water rather oftener than usual, and sometimes with difficulty; and that he had a constant shooting pain in the perinæum, which was increased after micturition. He had also occasionally a sense of "weakness" in the right lumbar region. Urine of natural appearance; and functions natural.

On examining the substances above mentioned, one was discovered of a globular shape, and about one and a quarter inches in circumference, evidently an hydatid, of the genus Acephalocyst. It was filled with a transparent fluid, having floating in it another very small hydatid, which gravitated in the surrounding fluid. The remainder of the substances consisted of the coats of seven or eight hydatids which had burst, and which, when filled with water, varied in bulk from the size of a pea to that of a pigeon's egg.

He stated that, seven months ago, he got a "bad cold," and suffered from pain above the right hip, and in the perinæum; and that, five months ago, a blister was applied, which removed the pain above the ilium, but that he still feels occasional uneasiness there. About a month ago, he passed several hydatids, which caused some obstruction to the flow of urine, but no more appeared until three days ago, although during the last month he has had constant pain in the perinæum, apparently near the neck of the bladder.

He was ordered to take diluted muriatic acid, twelve minims three times a day.

16th. Another hydatid has been passed (burst). The pain is nearer the end of the penis.

24th and 25th. Two more hydatids passed, which obstructed the urine for some time. No pain in the perinæum now; it is generally felt six or seven hours before the hydatid is expelled.

June 3. No more hydatids have appeared. Complains only of weakness in the back and hip.

The above case is interesting, from the extreme rarity of its occurrence. Dr. Craigie says, that "the uterus is the only cavity, with mucous surface, in which inspection shows that hydatids have been found;" and there can be no doubt that, in this case, they were formed in the kidneys, and probably increased in size after their descent into the bladder.

The following account of the post-mortem appearances in one of the few instances of the kind on record, is taken from the Philosophical Transactions for 1687. Dr. Tyson, in stating what was observed in the bladder, says, "therein, upon apertion, we discovered a very strange sort of cystes or bags, of the exact figure of eggs, of several dimensions, some larger than goose-eggs, others as big as hen-eggs, to the number of twelve in all; and about eight of them whole and repleat with a limpid serum; all of them loose and free, without the least adhæsion, either to one another or to the coat of the bladder; nor could we imagine that this miserable patient could possibly make any water but what happened upon the breach of some of these watery tumors, when the bladder was crouded beyond its dimensions. The vreters were of the largeness of the small gutts in children, so that they could easily admit two fingers into their cavity. One of the vesiculæ being opened, had a large cluster of small ova as big as grapes, all repleat with liquor. All the rest contained nothing but serum." Two small ova were observed at the entrance of each ureter, having descended from the kidneys.—*The Liverpool Medical Journal, July.*

CASE OF AMAUROSIS CURED BY STRYCHNINE.

Edward Sale, æt. twenty-eight, a stonemason, applied to Mr. Neill, at this Institution, April 17th. Complained of diminished vision in the left eye, with confusion from diplopia and muscæ-volitantes; pain over the brow, and much occasional pain of the head; the pupil is dilated, but slightly moveable.—Pil. Hyd. gr. iv. night and morning, and the drop of the Vin. Opii to be applied in the morning.

26th. Mouth sore; great pain of head. To be cupped to ℥xiv. on the nape of the neck, and have Sulph. Magnesia one oz. and one gr. of the Tartarised Antimony.

29th. Head relieved, but vision not at all improved. A blister to be applied to the nape of the neck.

May 1st. Half grain of strychnine dusted over the blistered surface.

3d. Thinks his sight was better after the application. One gr. applied.

4th. A sensation of bitterness in the mouth, and since two hours after the last application has had a feeling of suffocation, as if from sulphur, but vision improved. One gr. dusted on.

5th. The left eye is quite well; but the dimness has attacked the right one; with it he perceives objects double. One gr.

6th. One gr. repeated.

7th. Double vision gone.

8th. One gr., then discontinue.

15th. Discharged cured.—*Ibid., August.*

